# Cognitive control and emotional response in attention-deficit/ hyperactivity disorder comorbidity with disruptive, impulse-control, and conduct disorders

**DOI:** 10.1186/s12888-021-03221-2

**Published:** 2021-05-04

**Authors:** Yuncheng Zhu, Li Liu, Daoliang Yang, Haifeng Ji, Tianming Huang, Lianxue Xue, Xixi Jiang, Kaiyun Li, Lily Tao, Qing Cai, Yiru Fang

**Affiliations:** 1 Shanghai Hongkou Mental Health Center, Shanghai, 200083 China; 2 Clinical Research Center, Shanghai Mental Health Center, Shanghai Jiao Tong University School of Medicine, Shanghai, 200030 China; 3 Shanghai Changning Mental Health Center, Shanghai, 200335 China; 4 University of Jinan, Shandong Province, Jinan, 250022 China; 5 Key Laboratory of Brain Functional Genomics (MOE & STCSM), Shanghai Changning-ECNU Mental Health Center, Institute of Cognitive Neuroscience, School of Psychology and Cognitive Science, East China Normal University, Shanghai, 200062 China; 6 CAS Center for Excellence in Brain Science and Intelligence Technology, Shanghai, 200031 China; 7 Shanghai Key Laboratory of Psychotic disorders, Shanghai, 201108 China

**Keywords:** Attention-deficit/hyperactivity disorder, Disruptive, impulse-control, and conduct disorders, Neural network, Cognitive control, Stroop effect

## Abstract

**Background:**

This study investigated cognitive and emotional functioning in children and adolescents with attention-deficit/hyperactivity disorder (ADHD) and disruptive, impulse-control, and conduct disorders (DICCD).

**Methods:**

Thirty patients with ADHD, 26 with DICCD, 22 with ADHD+DICCD were recruited from the outpatient department of Shanghai Changning Mental Health Center, plus 20 healthy controls (HC). Differences between the groups in cognitive and emotional functioning were examined using Golden’s Stroop and Emotional Stroop tests. For Emotional Stroop Mean reaction time (RT) of positive word (POS) and negative word (NEG) with color congruence (C) or incongruence (I) were recorded as POS-C, POS-I, NEG-C and NEG-I, respectively.

**Results:**

For Golden’s interference scores (IGs), both errors and RTs in the ADHD group were higher than in the other groups. Longer mean RTs of POS-C, POS-I, NEG-C and neural word (NEU) of the ADHD group, and NEG-I of ADHD+DICCD and DICCD groups were observed compared to HC. After 12 weeks of methylphenidate treatment, differences between ADHD subgroups and HC on Golden’s Stroop RT disappeared, but differences in Golden’s Stroop errors and Emotional Stroop mean RTs remained. The ADHD+DICCD group showed longer mean RTs in NEG-C, NEG-I and NEU of the Emotional Stroop test than the ADHD group.

**Conclusions:**

Our study shows that regardless of emotional responding, deficit in cognitive control is the core symptom of ADHD. However, emotionally biased stimuli may cause response inhibitory dysfunction among DICCD with callous-unemotional traits, and the comorbidity of ADHD and DICCD tends to account for the negative emotional response characteristic of DICCD. These deficits may be eliminated by medication treatment in ADHD, but not the ADHD with comorbid DICCD. Our results support the notion that ADHD with comorbid DICCD is more closely related to DICCD than to ADHD.

## Background

Attention-deficit/hyperactivity disorder (ADHD) is a common neurodevelopmental disorder in children and adolescents that comprises core symptoms of high levels of inattention, motor hyperactivity, and impulsivity [1]. ADHD ranks among the highest of children’s mental disorders, with a prevalence of 6.26% in China, with difficulties often continuing into adulthood [2]. Furthermore, it is estimated that comorbid disruptive, impulse-control, and conduct disorders (DICCD) occurs in 20 to 78% of cases [3, 4]. The treatment difficulty in ADHD is currently still unresolved, resulting in poor prognosis of the disorder. These issues have so far not received adequate attention. Consequently, the low rate of treatment and high rate of missed diagnosis of ADHD have become a serious public health problem worldwide.

Previously, ADHD and DICCD were classified under attention deficit and destructive behavior in Diagnostic and Statistical Manual of Mental Disorders, Fourth Edition (DSM-IV) [5]. It was only until the release of DSM-5 in 2013 that ADHD was first defined as a neurodevelopmental disorder, whereas conduct disorder (CD) and oppositional defiant disorder (ODD) were still catalogued under DICCD. We investigated ADHD, CD and ODD which are the most common diagnosed behavioural disorders [6]. We did not put other disorders under DICCD into consideration, such as intermittent explosive disorder, kleptomania or pyromania, for the low probability of morbidity. A special statement made by DSM-5 is that disassembling the three disorders does not deny the high rate of comorbidity among them, but highlights the essence of the neurodevelopmental defects of ADHD [7]. However, the diagnosis of ADHD is still mainly based on symptomatology and subjective medical history provided by parents, without objective biomarkers.

Neuropsychiatric research not only emphasizes the discussion of symptomatology, but also the neuroscientific perspective of the occurrence and development of ADHD [8]. It is worth noting that attention deficits, impulsivity and hyperactivity are the core symptoms listed in DSM-5, whereas irritability and emotional instability are only mentioned as related traits of ADHD. In addition, cognitive problems – such as executive dysfunction and other cognitive processing impairments – are also major pathological features of ADHD, under this diagnostic system [9]. Executive function (EF) comprises two domains, inhibition and metacognition [10] or was outlined as inhibition and cognitive flexibility [11]. The former encompasses the inhibitive ability in motor, verbal, cognitive, and emotional activities. Deficits in this domain contribute to deficits in the domain of cognitive flexibility, including nonverbal working memory (motor activity), verbal working memory (verbal activity), planning and problem-solving (cognitive activity), and emotional self-regulation (emotional activity). Herein, we hypothesized that the pathogenicity of emotion is also closely associated with ADHD. Emotion-related problems in DICCD (ODD/CD) are associated with dysfunctions in two distinct neurocircuits, one for response inhibition and the other for emotional responding [12]. There is neurobiological evidence to support the inclusion of the emotional domain in the core ADHD phenotype [13]. In addition, Blair, R. J. et al. [14] found aspects of cognitive control are also impaired in patients with conduct problems (ODD and CD). The above neuroscientific findings have been supported in clinical data, namely the high comorbidity of ADHD and DICCD.

A relevant theoretical framework is the EF model of ADHD, which is divided into two systems, “cool” and “hot” EF (CEF and HEF) [15]. CEF, also known as “pure” cognitive processing [16], is a top-down process of cognitive control, emotion-independent and logically-based. It is required to solve abstract and contextualized problems including adaption, task-switching, cognitive control or strategic change [17]. In contrast, HEF includes both top-down and bottom-up processes [18], involving feedback of cognitive control in emotional responses and emotional decision-making, including emotion, desires, motivation, and rewards. It is required when an individual is making choices with potentially rewarding or aversive consequences. Since cognitive processes interact, non-emotional and emotional information input are usually received simultaneously. CEF activates in emotionally neutral contexts to HEF needed for the reversal of motivationally significant tendencies [15].

The pathogenicity of emotion is also closely associated with ADHD. Several theoretical models of ADHD [19, 20] acknowledge that several related but distinct neural pathways lead to ADHD, among which a cognitive/inhibitory control pathway and an emotional/motivation pathway co-exist. There is strong evidence of meta-analysis that abnormalities in the amygdala are specific for ODD/CD, irrespective of the presence of ADHD comorbidity [21]. Studies suggesting that ODD/CD are driving cognitive problems in children with ADHD [22]. After treatment, a correlation exists between methylphenidate (MPH)-related improvement in ADHD symptoms and higher empathy in children with ADHD, but not for those comorbid with DICCD [23]. Based on the above, emotional neuropsychology testing should be able to distinguish emotional processes from non-emotional processes [24]. If confirmed, cognitive control and emotional responding may play different roles in superposition for callous-unemotional (CU) traits of ADHD [25]. Assessment of emotional responding (HEF) and cognitive control (CEF) can be distinguished [26].

The Stroop test is the classic paradigm for CEF [27]. In addition, we also employed the Emotional Stroop test for testing both CEF and HEF. On the basis of the original Stroop task, emotion words in the lexicon of the subjects were presented in a color that was either congruent or incongruent to its emotional valence. The main purpose of the present study was to find assess differences in EF among ADHD, DICCD, and comorbid groups using the Stroop tests (classic and emotional versions). Therefore, as a secondary purpose, we attempted to identify the effect of MPH on ADHD and on ADHD+DICCD after 12 weeks of treatment, to determine whether CEF or HEF is responsible for treatment resistance. All patients received general psychotherapy even if they were not on medication.

## Methods

### Study design

All subjects were assessed using the Golden’s Stroop Test and Emotional Stroop Test to evaluate cognitive control and emotional responding. The task had been tested and validated in children [28, 29]. Data at baseline and after 12 weeks of treatment were collected. The treatment was started after the first evaluation.

### Sample size calculation

Sample size was calculated using PASS 11 software. Significance level *α* was set to 0.05 and power of test 1-*β* was 0.8. We used a completely random design to compare the mean of multiple samples for sample size estimation. The calculations showed that a sample of 80 met the minimum sample size required.

### Setting

This study was approved by the Institutional Ethical Committee for clinical research of Shanghai Changning Mental Health Center, Shanghai, China. All subjects were Han Chinese, and parental consent was obtained prior to participation. Written informed consent was provided in accordance with the *Declaration of Helsinki*.

All patients were from the outpatient department of Shanghai Changning Mental Health Center. There were three groups of patients – ADHD (*n* = 30), DICCD (*n* = 26, ODD/CD = 19/6) and ADHD+DICCD (*n* = 22, ODD/CD = 16/6) – plus 20 healthy controls (HC).

The inclusion criteria were: (1) age between 9 and 16 years, pupils in grade three or above; (2) a diagnosis of ADHD or DICCD based on DSM-5 [7]; and (3) right-handed. The exclusion criteria were: (1) comparatively low IQ (< 80) as determined by Wechsler Intelligence Scale for Child-Fourth Edition (WISC-IV); (2) color blindness; (3) abnormal eyesight or corrected visual acuity; (4) a history of craniocerebral lesion, surgical trauma or birth with neonatal asphyxia; (5) congenital somatic diseases and genetic diseases; (6) any other mental disorders comorbidity; and (7) any other medications.

**Fig. 1:**
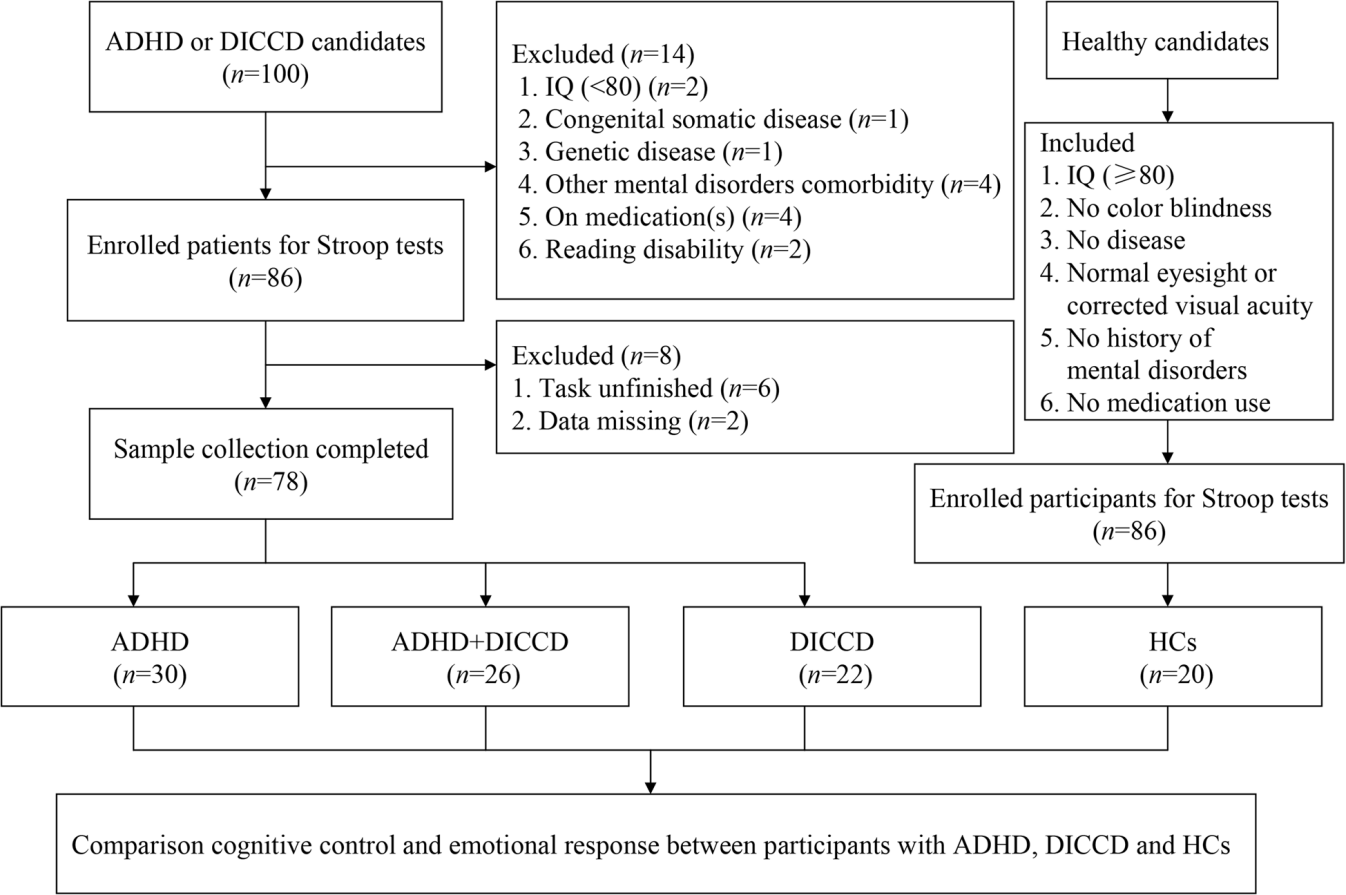
Flowchart of screening process and data classification

Diagnostic evaluation was performed by two experienced pediatric psychiatrists. Patients with suspected diagnosis or disagreement after consultation were not included in this study. HC, who were matches on age, IQ, sex, and educational background to patients, were recruited from primary and middle schools in Shanghai. HCs were screened by a psychiatrist of our team on ADHD, ODD and CD symptoms ahead of the study. See Fig. 1 for a flow diagram of sample selection.

Under MPH condition - CONCERTA (methylphenidate HCl) Extended-release Tablets- (18 mg/d dose, the minimum dose of tablet in Chinese pharmaceutical market), eighteen ADHD patients and 17 ADHD+DICCD patients who received the medication agreed to participate in the neuropsychological tests again after 12 weeks of treatment. The therapeutic regimen was not compulsory as our psychiatrists gave their patients to choose pharmacotherapy or general psychotherapy after being fully informed. We carry out a hotline to follow up throughout the process.

### Variables and data sources

#### Wechsler intelligence scale for child-fourth edition (WISC-IV)

WISC-IV [30] was used to evaluate IQ of children at 6–16 years old. The scale measured verbal comprehension, perceptual reasoning, working memory, processing speed, general cognitive ability and cognitive efficiency. The average IQ is 100, which is used to illustrate the overall cognitive abilities of children. Higher IQ indicates the higher overall cognitive ability.

#### Conners parents symptom questionnaire (PSQ)

PSQ [31] assesses symptom severity for DICCD and ADHD, and consisted of 48 items and 6 subscales: Conduct problem (12 items), Difficulties in learning (4 items), Psychosomatic disorders (5 items), Impulsivity/Hyperactivity (4 items), Anxiety (4 items) and Conners Index of Hyperactivity (CIH) (10 items). Each item requires a rating one a 4-point scale: from 0 = not true at all to 3 = very much true. Scores were converted to T scores based on sex and age of the child, with a score of > 65 indicating clinically elevated symptoms [32].

#### Golden’s Stroop test

In Golden’s Stroop color and word test [33], 126 words were randomly arranged in 14 × 9 (rows x columns). The test consisted of three parts: part A involved naming color patches (red, blue, green, or yellow patches); part B involved reading color words printed in black (“red”, “blue”, “green”, or “yellow”); part C required naming the color that the words are printed in, which was incongruent with what the word says (e.g., the word “red” printed in blue). Each part was followed by a 60 s rest interval, with a “+“presented for 100 ms before the next part began. The participants were instructed to respond as quickly and as accurately as possible by pressing the corresponding button. Stimuli were presented one-by-one. Reaction time (RT) and errors were recorded. Golden’s Stroop interference score (IG) was derived using the formula C - [(A * B)/ (A + B)] [34]. A higher IG indicates more severe deficit of cognitive control. If participants were fatigued during the task, rest time was extended. All participants completed the test.

#### Emotional Stroop test

In the Emotional Stroop test [29], 60 words selected from a Chinese thesaurus were divided into three categories of 20 words each: positive words (POS), negative words (NEG), and neutral words (NEU) [28]. The words of the Chinese thesaurus had been tested by Yufeng Zhen in her Chinese dissertation (An experimental study of Emotional Stroop Effect in Positive Stimulus) where frequency of words, stroke number and valence of words were validated. The positive words were sweet, passion, romance, happiness, pleasure, peace, joy, lenience, sincerity, tranquility, wish, pride, smartness, excitement, alacrity, purity, honest, briskness, luck, and self-confidence. The negative words were of shame, flinch, weeping, panic, anxiety, decadence, dreariness, weakness, mourning, depression, greed, annoyance, embarrassment, disappointment, sadness, timidity, distress, abnormality, melancholy, and complaint. The neutral words were road, wall, territory, hotel, building, cave, apartment, island, wave, mileage, scene, tunnel, village, stage, capital, boundary, market, sapling, terrace, and field. One each trial, a “+“first appeared for 100 ms. The words then appeared in one of two colors (red or blue) randomly in the middle of screen. Participants were instructed to press the “F” key with their left index finger when red appears, and press the “J” key with their right index finger when blue appears. Stimuli were presented one-by-one. The program defines red to be congruent with POS and blue to NEG. The mean RT, POS words and NEG words with color congruence (C) or incongruence (I) were recorded as POS-C, POS-I, NEG-C or NEG-I. For NEU words only RT was recorded. The test was divided into three blocks, with 40 trials in each, and rest period between blocks of 60 s. Before recording, our researchers confirmed reading ability of participants by reading these words and doing a block training. The procedure would have been terminated if they did not have a reading mastery. Compared to HC, mean RTs indicated severity of cognitive control deficit and emotional responding deficit.

### Bias

All data were evaluated by normality test and test of homogeneity of variance. Apart from the errors of Golden’s Stroop IG, the remaining variables of both Stroop tests had normal distribution and equal assumed variance. Programmer designed two emotional Stroop tests to balance the left and the right choice. Only one was kept in case that confuses participants if the rule changed during the study.

### Quantitative and qualitative variables

The quantitative variables were expressed as mean ± standard deviation (SD) and qualitative variables as *n* (%).

### Statistical analysis

SPSS 22.0 software was used to carry out statistical analyses. One-way ANOVA was conducted for variables with normal distribution and homogeneity of variance, and Kolmogorov-Smirnov Z Test or Kruskal-Wallis H Test was carried out for variables with skewed distribution or heterogeneity of variance like psychosomatic disorders, anxiety and errors of Stroop. Analysis of covariance was further conducted for the comparison of baseline and follow-up values of the Emotional Stroop test, with the difference in Golden’s Stroop RT IG before and after treatment as the covariable *(see details in the supplementary material)*. Differences between groups were analyzed using Post Hoc tests. The sex ratio was checked by chi-square. Diagnostic classification uses four values as category variables (1 = ADHD, 2 = ADHD+DICCD, 3 = DICCD, 4 = HC). A two tailed *p*-value < 0.05 was predetermined as statistically significant. Bonferroni correction for multiple comparisons was applied, and the level was p = p’/c, c (number of pairwise comparisons) = (k (k-1)/2). After normal transformation if necessary, the non-normally distributed data were analyzed with statistical disposal for effect sizes employed by *η*^*2*^.

### Statistical analytic plan

We compared differences in Golden’s Stroop test and Emotion Stroop test variables among the ADHD, ADHD+DICCD, DICCD and HC groups at baseline level as primary outcomes. For secondary outcomes, differences in those variables were compared between ADHD and ADHD+DICCD groups at follow-up (after 12 weeks of treatment), and differences between baseline and follow-up (cohort design) were compared within the ADHD group.

## Results

### Demographic and clinical information

The four groups of participants (three groups of patients plus HC) did not differ in sex ratio (*χ*^2^ = 2.734, *p* = 0.434), age (*F* = 2.302, *p* = 0.082), and IQ (*F* = 1.007, *p* = 0.393). See Table 1.

**Table 1:** Comparison of demographic variables in ADHD, ADHD+DICCD, DICCD and HC groups

	ADHD (*n* = 30)	ADHD+DICCD (*n* = 26)	DICCD (*n* = 22)	HC (*n* = 20)	*F/χ2*	*p*
Age (years)	12.7 ± 2.5	11.7 ± 2.9	11.1 ± 2.3	12.8 ± 2.6	2.302	0.082
IQ	99.9 ± 9.5	97.5 ± 10.9	96.7 ± 7.6	101.4 ± 11.8	1.007	0.393
Sex [n (%)]						
Male	23(76.7%)	15(57.7%)	15(68.2%)	15(75.0%)	2.734	0.434
Female	7(23.3%)	11(42.3%)	7(27.3%)	5(25.0%)		

Note: *ADHD* Attention-deficit/ hyperactivity disorder; *ADHD+DICCD* Attention-deficit/ hyperactivity disorder with comorbid disruptive behavior disorder, *DICCD* Disruptive behavior disorder, *HC* Healthy controls

**Fig. 2:**
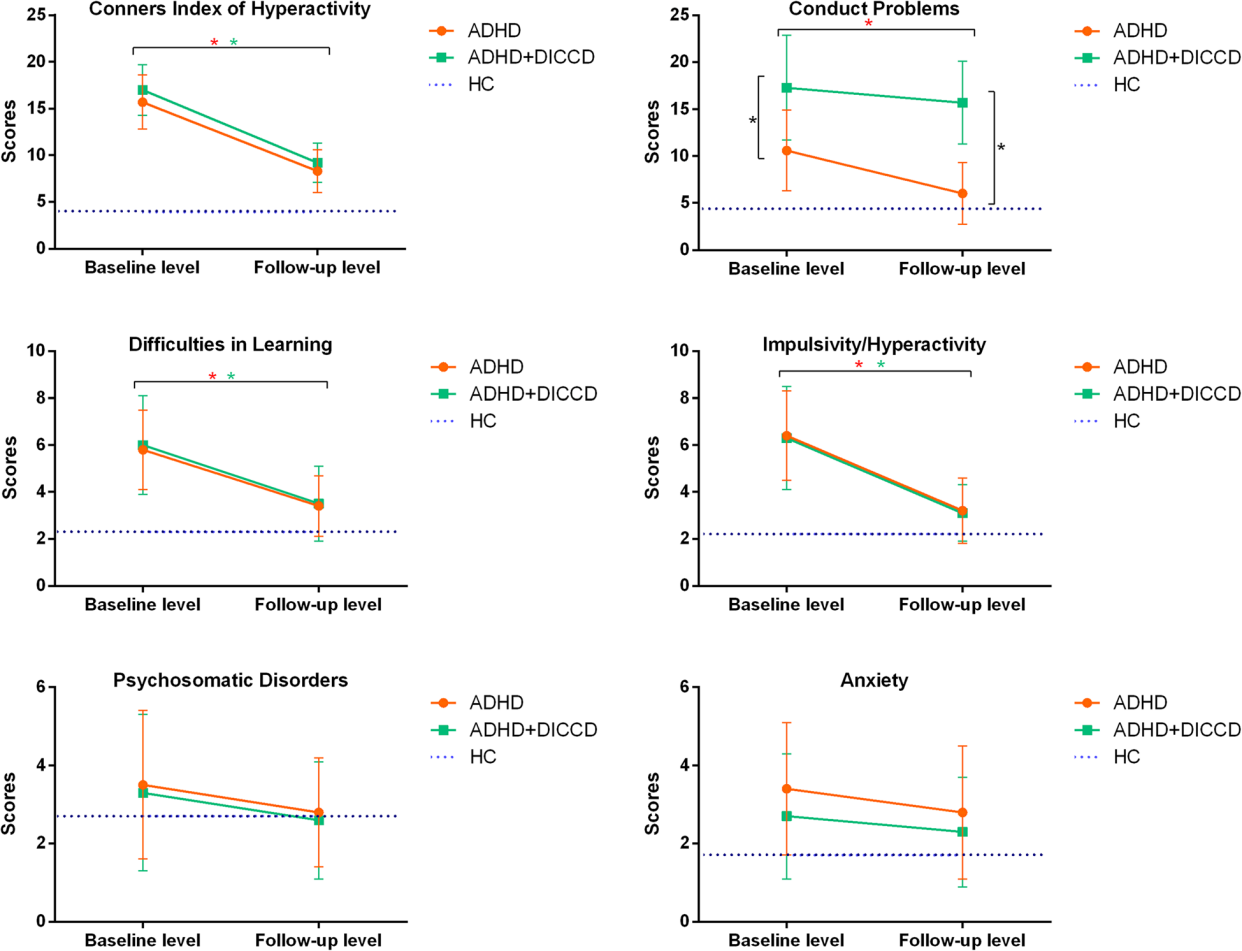
Comparison of the subscles of PSQ between baseline and follow-up ADHD subgroups. * means the statistical difference

The statistical difference scores of PSQ factors among the groups included CIH, Conduct Problems, Difficulties in Learning, Impulsivity/Hyperactivity, and Anxiety (*F/H*[11.58, 132.8], *p’*s < 0.05), and they were further analyzed using Post Hoc comparisons for subgroups. The scores of CIH, Impulsivity/ Hyperactivity in the DICCD group were significantly lower than the ADHD and ADHD+DICCD groups but higher than the HC group. The scores of Conduct Problems in the ADHD group were lower than the ADHD+DICCD and DICCD groups but higher than the HC group. The scores of Difficulties in learning for all three disorder groups were higher than the HC group, but there was no statistical difference among the disorder groups. Compared to the HC group, scores of Anxiety of the ADHD group were significantly increased. The values were shown in Table 2 and Fig. 2.

**Table 2:** Comparison of scores of Conners parent symptom questionnaire among groups at baseline level

	ADHD (*n* = 30)	ADHD+DICCD (*n* = 26)	DICCD (*n* = 22)	HC (*n* = 20)	*F/H*	*p*	Post Hoc
Conners Index of Hyperactivity	15.7 ± 2.9	17.0 ± 2.7	10.5 ± 2.1	3.9 ± 1.7	132.8	< 0.001	1,2 > 3 > 4
Conduct Problems	10.6 ± 4.3	17.3 ± 5.6	17.1 ± 4.2	4.4 ± 2.0	44.41	< 0.001	2,3 > 1 > 4
Difficulties in Learning	5.8 ± 1.7	6.0 ± 2.1	4.3 ± 1.4	2.3 ± 1.2	22.80	< 0.001	1,2,3 > 4
Psychosomatic Disorders	3.5 ± 1.9	3.3 ± 2.0	2.6 ± 1.7	2.7 ± 1.5	5.045	0.169	
Impulsivity/Hyperactivity	6.4 ± 1.9	6.3 ± 2.2	4.3 ± 1.7	2.2 ± 1.0	27.52	< 0.001	1,2 > 3 > 4
Anxiety	3.4 ± 1.7	2.7 ± 1.6	2.5 ± 1.7	1.7 ± 1.4	11.58	0.009	1 > 4

Note: *ADHD* Attention-deficit/ hyperactivity disorder, *ADHD+DICCD* Attention-deficit/ hyperactivity disorder with comorbid disruptive behavior disorder, *DICCD* Disruptive behavior disorder; *HC* Healthy controls

### The measurements of Golden’s Stroop test and emotional Stroop test

In the Golden’s Stroop test, IG errors (*H* = 15.93, *p* = 0.003) and IG RT (*F* = 3.505, *p* = 0.044) yielded differences between groups. Post Hoc showed that IG errors in the ADHD group were higher than the ADHD+DICCD, DICCD and HC groups, and the IG RT was higher in the ADHD group compared to the HC group.

In the Emotional Stroop test, the mean RT exhibited statistical difference among the groups (*F/H*[3.495, 4.279], *p’*s < 0.05). Post Hoc tests showed that the longer POS-C of ADHD was observed compared to HC while longer POS-I, NEG-C and NEU of ADHD and ADHD+DICCD were observed compared to HC. However, longer mean RTs of NEG-I of ADHD+DICCD and DICCD were observed compared to HC while it yielded no difference between ADHD and HC. See Table 3.

**Table 3:** Comparison of Golden’s Stroop test and Emotion Stroop test among groups at baseline level

	ADHD (*n* = 30)	ADHD+DICCD (*n* = 26)	DICCD (*n* = 22)	HC (*n* = 20)	*F/H*	*p*	*η*^*2*^	Post Hoc
Golden’s Stroop IG								
Errors of Stroop	5.5 ± 3.8	2.9 ± 2.6	2.4 ± 1.7	2.0 ± 1.9	15.93	< 0.001	0.337	1 > 2, 3, 4
Time of Stroop (s)	180.0 ± 44.4	157.6 ± 44.9	147.7 ± 48.2	143.7 ± 39.0	3.505	0.018	0.101	1 > 4
Emotional Stroop Mean RT (ms)								
POS-C	808.4 ± 196.5	781.4 ± 146.4	796.9 ± 82.3	672.7 ± 147.7	3.582	0.017	0.103	1 > 4
POS-I	840.0 ± 243.0	816.7 ± 190.8	871.8 ± 110.0	684.3 ± 189.2	3.771	< 0.013	0.103	1, 3 > 4
NEG-C	805.6 ± 209.8	769.8 ± 163.6	817.2 ± 90.9	656.0 ± 149.0	4.279	< 0.007	0.197	1, 3 > 4
NEG-I	864.6 ± 241.8	899.6 ± 223.9	934.7 ± 125.1	729.9 ± 218.2	3.765	< 0.013	0.120	2, 3 > 4
NEU	813.9 ± 206.7	791.7 ± 162.6	815.0 ± 94.7	675.3 ± 155.4	3.495	< 0.019	0.100	1, 3 > 4

Note: Golden’s Stroop IG, interference score of Golden’s Stroop test; Emotional Stroop MRT, mean reaction time of Emotional Stroop test; *POS-C* Positive word-color congruence, *POS-I* Positive word-color incongruence, *NEG-C* Negative word-color congruence; (C), *NEG-I* Negative word-color incongruence, *NEU* Neutral word; a, attention-deficit/ hyperactivity disorder; b, attention-deficit/ hyperactivity disorder with comorbid disruptive behavior disorder; c, disruptive behavior disorder; d, healthy controls

### Comparison of the variables of PSQ and Stroop test between baseline and follow-up ADHD subgroups

After 12 weeks of MPH treatment (18 mg/d dose), there were still significant differences in the subscale scores of PSQ among the three groups (*F* [4.200, 40.70], *p’*s < 0.05), except for Psychosomatic Disorders (*H* = 0.357, *p =* 0.837) and Anxiety (*H* = 3.807, *p =* 0.149) subscales. In addition, the only significant difference in PSQ was Conduct Problems between ADHD subgroups (*t* = 7.436, *p* < 0.001) at follow-up. See Table 4 and Fig. 2.

**Table 4:** Comparison of PSQ and Stroop test before and after 12 weeks between ADHD subgroups and HC group

	Baseline		*t /Z*	*p*	Follow-up		*t /Z*	*p*	ADHD (baseline vs follow-up)		ADHD+DICCD (baseline vs follow-up)		Follow-up (ADHD VS ADHD+DICCD VS HC)	
	ADHD (*n* = 30)	ADHD+DICCD (*n* = 26)			ADHD (*n* = 18)	ADHD+DICCD (*n* = 17)			*t /Z*	*p*	*t /Z*	*p*	*F/H*	*p*
PSQ														
CIH	15.7 ± 2.9	17.0 ± 2.7	1.595	0.117	8.3 ± 2.3	9.2 ± 2.1	1.260	0.217	9.336	< 0.001	9.913	< 0.001	36.42	< 0.001
Conduct Problems	10.6 ± 4.3	17.3 ± 5.6	5.071	< 0.001	6.0 ± 3.3	15.7 ± 4.4	7.436	< 0.001	4.141	< 0.001	1.017	0.315	40.70	< 0.001
Difficulties in learning	5.8 ± 1.7	6.0 ± 2.1	0.381	0.704	3.4 ± 1.3	3.5 ± 1.6	0.163	0.872	4.987	< 0.001	4.159	< 0.001	4.200	0.020
Psychosomatic Disorders	3.5 ± 1.9	3.3 ± 2.0	0.536	0.592	2.8 ± 1.4	2.6 ± 1.5	0.304	0.761	1.217	0.224	0.870	0.384	0.357	0.837
Impulsivity/Hyperactivity	6.4 ± 1.9	6.3 ± 2.2	0.037	0.971	3.2 ± 1.4	3.1 ± 1.2	0.240	0.812	6.087	< 0.001	6.090	< 0.001	4.736	0.013
Anxiety	3.4 ± 1.7	2.7 ± 1.6	1.606	0.108	2.8 ± 1.7	2.3 ± 1.4	0.691	0.490	1.235	0.217	0.603	0.546	3.807	0.149
Golden’s Stroop IG														
Errors of Stroop	5.5 ± 3.8	2.9 ± 2.6	2.657	0.008	3.9 ± 2.4	2.4 ± 1.8	1.833	0.067	1.405	0.160	0.215	0.829	7.100	0.029
Time of Stroop (s)	180.0 ± 44.4	157.6 ± 44.9	1.868	0.067	161.9 ± 32.2	144.5 ± 30.7	1.526	0.137	1.628	0.111	0.957	0.344	1.547	0.222
Emotional Stroop MRT (ms)														
Positive-congruence	808.4 ± 196.5	781.4 ± 146.4	0.587	0.560	811.6 ± 64.2	826.7 ± 36.6	0.860	0.397	0.082	0.935	1.506	0.143	17.50	< 0.001
Positive-incongruence	840.0 ± 243.0	816.7 ± 190.8	0.402	0.689	867.4 ± 62.5	900.8 ± 41.0	1.861	0.072	0.585	0.562	2.173	0.038	18.32	< 0.001
Negative-congruence	805.6 ± 209.8	769.8 ± 163.6	0.717	0.476	789.5 ± 52.0	855.5 ± 41.2	4.144	< 0.001	0.400	0.691	2.551	0.017	20.72	< 0.001
Negative-incongruence	864.6 ± 241.8	899.6 ± 223.9	0.558	0.579	816.2 ± 47.1	986.2 ± 42.4	11.20	< 0.001	1.065	0.295	1.920	0.065	24.52	< 0.001
Neuter	813.9 ± 206.7	791.7 ± 162.6	0.449	0.655	773.8 ± 58.0	863.1 ± 39.9	5.275	< 0.001	0.998	0.325	2.144	0.040	17.15	< 0.001

Note: *PSQ* Conners Parents Symptom Questionnaire, *CIH* Conners Index of Hyperactivity, *IG* Golden’s interference score; *MRT* Mean reaction time

**Fig. 3:**
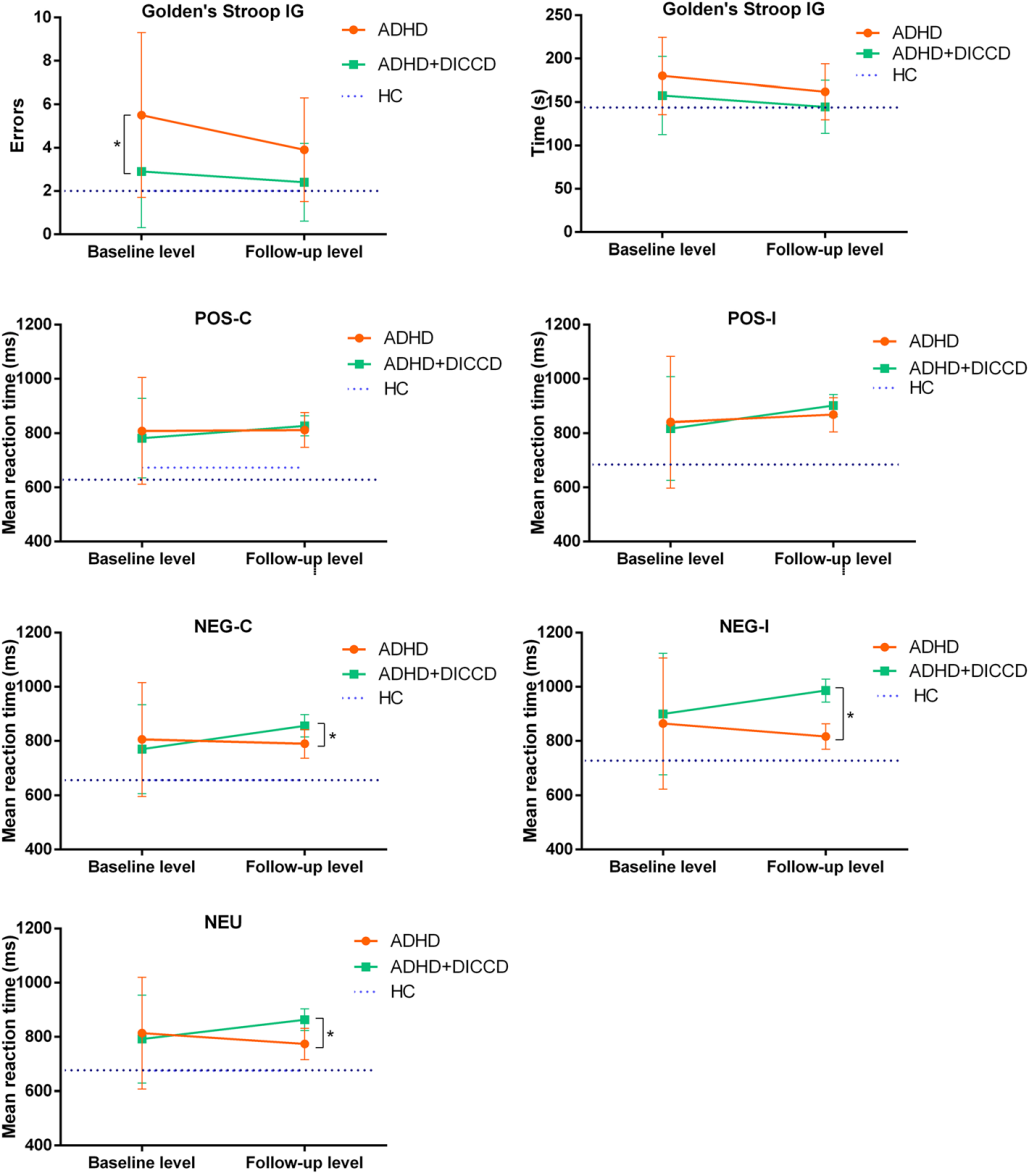
Comparison of the Stroop test between baseline and follow-up ADHD subgroups. * means the statistical difference

At follow-up, there were no statistical differences in the variables of Golden’s Stroop test between ADHD subgroups (*Z/t* = 1.833/1.526, *p’*s = 0.067/0.137). However, Emotional Stroop test showed that the mean RTs of NEG-C, NEG-I and NEU of ADHD+DICCD were longer compared to ADHD (*t* = 4.144/5.275/11.20, *p’*s < 0.001). See Table 4 and Fig. 3.

In the cohort design, there were no differences in the variables of Golden’s or Emotional Stroop test in the ADHD or ADHD+DBD groups between the first test and 12-week retest (*p*’s ≥ 0.05). No difference in Golden’s Stroop test RT was observed among the ADHD, ADHD+DBD and HC groups (*F* = 1.547, *p* = 0.222), but errors of Golden’s Stroop test for ADHD was higher than that for HC (*H* = 7.100, *p* = 0.029). For variables of Emotional Stroop test, statistical differences among the 3 groups were observed (*F* [17.15, 24.52], *p’s* < 0.001). Post Hoc tests showed that the mean RTs of NEG-I and NEU of ADHD+DICCD patients were significantly longer than those of ADHD patients and HC while the mean RTs of POS-C, POS-I, and NEG-C were significantly longer in the ADHD and ADHD+DICCD groups than those in the HC group. See Table 4 and Fig. 3.

## Conclusions

The present study investigated EF among ADHD, DICCD, and comorbid groups. Participants were assessed using the Stroop tests. The scores of PSQ exhibited good consistency with clinical diagnosis of ADHD with or without DICCD. The subscales (such as CIH, Impulsivity/Hyperactivity, and Conduct Problems) were able to distinguish between ADHD, DICCD and comorbidity. Moreover, the PSQ assessment of symptom reduction was also essential for the quantitative analysis of therapeutic effectiveness.

Evidence supports the use of extended-release MPH to improve symptoms of ADHD in adolescents. Psychosocial treatments were associated with inconsistent effects on ADHD symptoms and greater benefit for academic and organizational skills [35]. The primary pharmacologic effect of MPH is to increase central dopamine and norepinephrine activity, which impacts executive and attentional function [36]. Response inhibition is a critical executive function. AMPA receptors in the prefrontal cortex are involved in the effect of MPH on response inhibition in rats [37]. Additional MPH treatment study on response inhibition in adolescents is needed.

The errors of IG showed a statistical difference between the ADHD subgroups at baseline level (before treatment), which disappeared after the 12-week treatment, but the errors for the patient groups were still higher than those of the HC group. On the other hand, the RT of IG normalized to HC level at follow-up. Results also verify CEF deficit as a phenotype of ADHD, and that the function can be improved by MPH treatment. Although there were differences in Emotional Stroop test between ADHD children and typically-developing children at baseline, there were no differences between the ADHD subgroups.

Red has the additional meaning of Yang/positive/hot while blue implies Yin/negative/cold on the other hand. Yin and Yang are the two opposing principles in nature in the Chinese tradition. Therefore, red and blue were defined as “positive”and “negative” respectively, which is consistent with the theory of HEF and CEF. However, the significant differences were observed in the mean RT of NEG-I, and NEU among the ADHD subgroups. Our results suggest abnormal processes in negative emotional responding in the ADHD+DICCD group, which verifies our hypothesis that negative emotional responding of ADHD+DICCD is refractory even after MPH treatment. This may be a different phenotype of the EF of ADHD.

Firstly, we assessed CEF using a classic test for cognitive control. Our results indicated that cognitive control deficit is a core symptom of ADHD, regardless of whether emotional responding plays a role. ADHD patients undergo dysregulation of sustained and selective attention and behavioral traits, leading to the deficit of cognitive control within CEF [2].

Secondly, we speculated that the emotional impairment may be closely correlated with CU traits and violent tendencies [12]. In a study of CD, cognitive control under negative emotional stimulation is affected in patients with CD but not in HC [38], because the activation of prefrontal cortex in response to negative stimuli of aggressive traits is significantly reduced in adolescents with DICCD [39]. Due to the high rate of comorbidity of ADHD and DICCD, we may discover whether the mood dysregulation is the mechanism of ADHD [40]. To explain this possibility, we applied the concept of HEF in youth with ADHD symptoms.

At baseline, it was found that the negative emotional stimuli may impact the corresponding cognitive control via Stroop interference effect, but the interference does not seem to be neutral [38]. It has been shown that the deficit of HEF of emotional feedback not only contributes to the abnormal emotional responses in the ADHD group and the DICCD group, but also interferes with CEF implicated by the feedback in the cognitive control process. This suggests that monitoring and regulating processes within CEF (pure logic analysis like cognitive control) may involve different abilities than monitoring and regulating processes of HEF (psychological process driven by emotion) [27, 41].

The results of Golden’s Stroop test demonstrated that there was no significant difference between the DICCD and HC groups when emotional responding was not involved. Additionally, deficits in response inhibition and emotional responding were not observed in the ADHD+DICCD group, except for the NEG-I variable. After the inconsistency of emotional responses was added, the emotional effect on the DICCD group was significantly different from that of the HC group. In particular the NEG-I subunit, which was the most affected variable, did not differ between ADHD and HC at baseline, but differed for DICCD and ADHD+DICCD subgroups. In a previous Chinese study of ADHD comorbidity, ADHD+DICCD subgroup displayed better performance in naming colors and color words, and also had a tendency for shorter word interference time than pure ADHD group [42]. We conclude that ADHD, whether or not comorbid with DICCD, is closely associated with deficits in EF. ADHD+DICCD group showed significant EF deficit compared with the HC group, but the degree of executive dysfunction were less than pure ADHD group [42]. The combined results of Golden’s Stroop and Emotional Stroop tests suggest that bias in emotional stimuli may be responsible for CU traits of the DICCD patients, which produces over-suppression effects on the function of cognitive control [12]. Although abnormal processing interferes with emotional responding in the process of cognitive control, it was still suppressed by the core deficit of cognitive control in ADHD [43]. Therefore, differences in the Emotional Stroop test indicated that the emotional Stroop effect in DICCD was less affected than that in ADHD at baseline (before treatment).

In regard to Golden’s Stroop IG, there was also no difference between the ADHD+DICCD group and the DICCD group. Our evaluations showed that the cognitive control deficit of these two groups was less severe than ADHD patients. However, this result can not explain the normality of cognitive control of the DICCD. We suggest that the ADHD+DICCD group may have a different phenotype of neuropsychology. One explanation is that the CU traits of DICCD are more likely to reflect the clinical features of patients with comorbidities [44]. Blair et al. [14] also concluded that DICCD has no deficit in cognitive control. Another explanation is that patients with DICCD, who are long plagued by conduct problems, will exert more effort to behave appropriately, thus improving the results of the Stroop test [45]. If any true difference between groups were to exist at baseline, it would have to have a medium effect size for cognitive control and small effect sizes relative to the severity of deficit of cognitive control and emotional response in patients.

Lastly, we compared treatment effects on ADHD subgroups. MPH is the first-line pharmacotherapy for ADHD. It has been shown that MPH, which activates and normalizes ADHD neural network, is the most widely used prescription drug for ADHD [46]. However, the mechanisms underlying the pharmacological actions of MPH to core neuropsychological processes underlying the comorbidity of ADHD and DICCD remain unclear. Based on clinical guidelines, we retested the patients after 12 weeks of MPH treatment [47]. We found that the average errors of the Golden’s Stroop task reduced from 5.5 times to 3.9 times, though the errors were still more than typically-developing children. A previous study found improved cognitive control for patients treated with MPH compared with medication-naïve participants [48]. Consistent with previous findings, we found that the ADHD+DICCD group showed less severe deficit in cognitive control after treatment.

For emotional responding, however, a different pattern of results was observed. Compared to typically-developing children, the mean RTs of ADHD and ADHD+DICCD in the Emotional Stroop test were longer. Dysfunction among ADHD+DICCD was further increased. Although ADHD treatment improved Stroop test performance, indicated by reduced errors, the mean RT of Emotional Stroop among ADHD+DICCD yielded no improvement. The mean RT of ADHD+DICCD was significantly longer than ADHD and HC after treatment. ADHD and CU traits highlighted the importance of understanding the impact of conduct problems on cognitive and emotional functioning and psychopathology of youth. Children in the ADHD+DICCD group may be less likely to be normalized by MPH treatment, depending on the presence of CU traits in ADHD [49]. Another issue is that of diagnostic agreement between DSM and International Classification of Diseases (ICD), with the ICD-10 [50] system now officially used in China. In DSM-5, ODD and CD are two parallel diagnoses categorized under DICCD. In ICD-10, however, ODD is a subtype of CD. Therefore, we regard CU traits as a unique characteristic of DICCD, in accordance with ICD-10, rather than as a subgroup factor of CD as regarded in some studies. We assume that CU traits, which belong to the scope of HEF, may be responsible for treatment resistance.

CU traits (callous, uncaring, unemotional) are kinds of personality traits, and have been found to moderate functional impairment in ADHD. Specifically, functional impairment in ADHD are positively regulated by CU traits at low and moderate levels. For functional impairments in ODD, however, no such associations are observed [51]. Frick and Nigg [4] proposed that for CD, integrating CU traits into the diagnostic criteria would be a key method for improving classification and discrimination of ODD and CD. Whether CU traits affiliate to CD independently of DICCD is still in debate. Currently, CU traits are widely considered as an early marker of DICCD in Chinese psychiatry.

We verified the notion that ADHD comorbid with DICCD is more closely related to DICCD than to ADHD [52]. This may be due to the CU traits of DICCD, which can provide an explanation for discriminating ADHD and DICCD as two disorders in childhood and adolescence, and in which the more severe disorder, that is, DICCD, engulfs ADHD especially in neuropsychological terms. An obvious reason is that MPH is recommended as first-line treatment for ADHD rather than its comorbidity. Appropriate treatment will need to be individualized according to the patient’s specific neuropsychology. It has been well demonstrated that, even under the premise of controlling correlated predictive variables, CU traits still have a synergistic effect on related mental disorders [53]. It was also found medical interventions toward patients with CU traits encounter more difficulties than interventions toward those without [44].

The purpose of many clinical research endeavors is to draw a conclusion regarding differences between disorder and health. However, the exploration of such patterns tends to ignore the comparison between correlated neuropsychiatric disorders, forming isolated disease characteristic from the process of clinical diagnosis without considering the diversity and generality between disorders. Results were largely limited by bringing subjectivity and uncertainty to differential diagnosis. Given these considerations, our research team included ADHD, DICCD and comorbidity together in the current neuropsychological study to observe cognitive and emotional functioning among different patients.

There remain, however, some limitations in the current study. First, we used the knowledge from neuroscience in our research proposal [2, 8, 26, 28], but the current study did not correlate the neuropsychological results with that of functional neuroimaging studies. Second, our patients came from outpatient clinics, where the family socioeconomic and environmental factors were difficult to match, and thus cannot reflect the distribution of disorders in the whole population. Recruiting volunteers also has this selective bias.

To assess emotional responding of ADHD with comorbid DICCD, testing should be designed not only for specific affective symptoms, but as a standardized measuring tool for effectively screen carriers of symptoms. The effectiveness of classical neuropsychological tools in classifying different neuropsychological processes may be improved by integrating theoretical and empirical research findings.

## Data Availability

The data that support the findings of this study are available from SCMHC. Restrictions apply to the availability of these data, which were used under license for this study.

## Abbreviations

ADHD: Attention-deficit/hyperactivity disorder
DICCD: Disruptive, impulse-control, and conduct disorders
DSM-IV: Diagnostic and Statistical Manual of Mental Disorders, Fourth Edition
CD: Conduct disorder
ODD: Oppositional defiant disorder
EF: Executive function
CEF: “cool” executive function
HEF: “hot” Executive function
CU: Callous-unemotional
MPH: Methylphenidate
WISC-IV: Wechsler Intelligence Scale for Child-Fourth Edition
PSQ: Conners Parents Symptom Questionnaire
CIH: Conners Index of Hyperactivity
HC: Healthy controls
RT: Reaction time
IG: Golden’s Stroop interference score
POS: Positive words
NEG: Negative words
NEU: Neutral words
C: Congruence
I: Incongruence
SD: Standard deviation
